# Supplementary material for: The causal relationship between the human gut microbiota and pyogenic arthritis: a Mendelian randomization study
Source: Front Cell Infect Microbiol. 2024 Nov 26;14:1452480. doi: 10.3389/fcimb.2024.1452480 (PMC11629706; doi:10.3389/fcimb.2024.1452480)
Supplement: Supplementary file 1 [file DataSheet1.zip › mendelian test/ebi-a-GCST90027771.csv_forest.pdf]

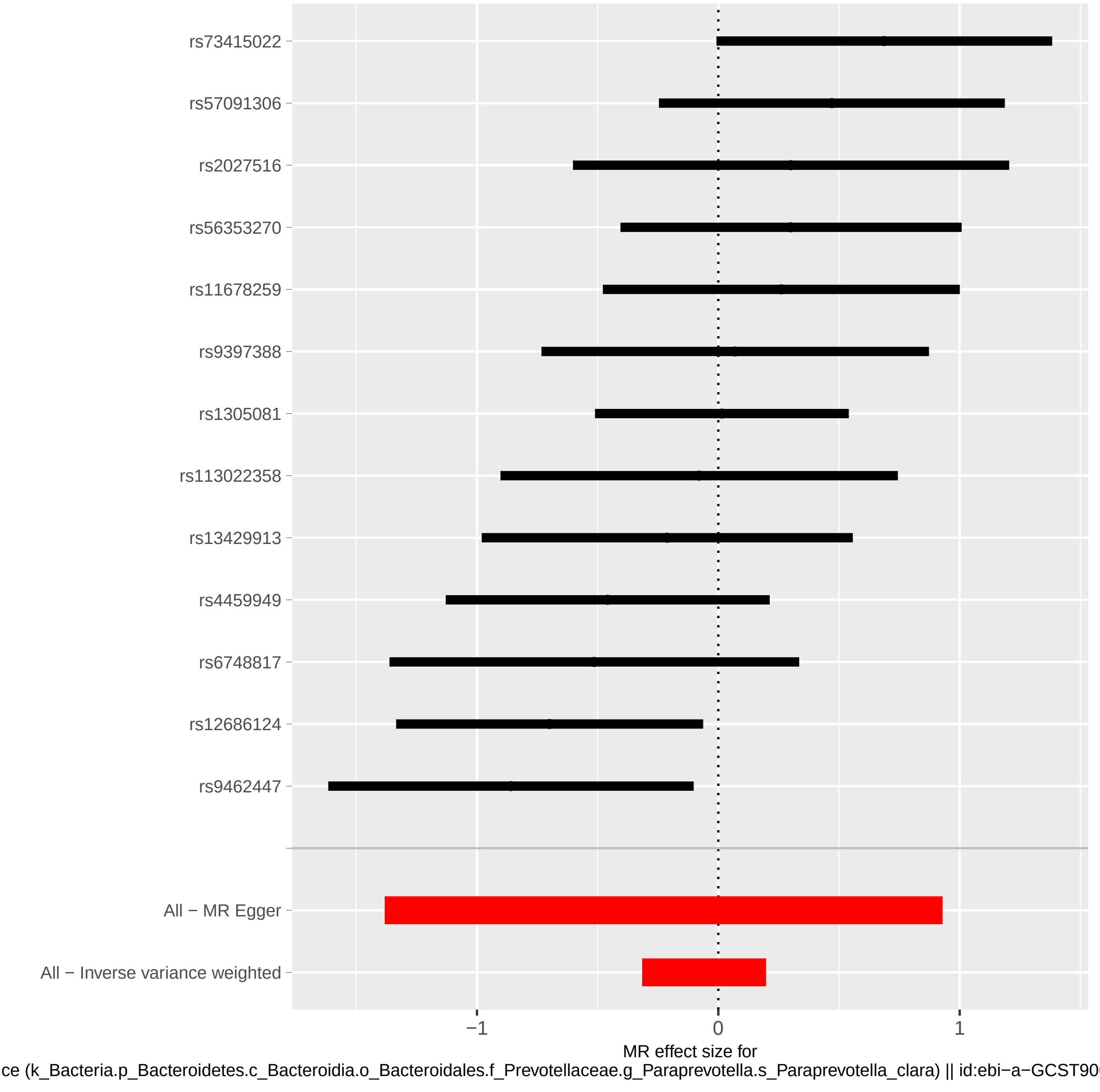

rs73415022
rs57091306
rs2027516
rs56353270
rs11678259
rs9397388
rs1305081
rs113022358
rs13429913
rs4459949
rs6748817
rs12686124
rs9462447
All − MR Egger
All − Inverse variance weighted
−1
0
1
MR effect size for
ce (k_Bacteria.p_Bacteroidetes.c_Bacteroidia.o_Bacteroidales.f_Prevotellaceae.g_Paraprevotella.s_Paraprevotella_clara) || id:ebi−a−GCST90
